# Ethylchloroformate Derivatization for GC–MS Analysis of Resveratrol Isomers in Red Wine

**DOI:** 10.3390/molecules25204603

**Published:** 2020-10-09

**Authors:** Elisa Di Fabio, Alessio Incocciati, Federica Palombarini, Alberto Boffi, Alessandra Bonamore, Alberto Macone

**Affiliations:** Department of Biochemical Sciences, “Sapienza” University of Rome, p.le A.Moro 5, 00185 Rome, Italy; elisa.difabio@uniroma1.it (E.D.F.); incocciati.1750499@studenti.uniroma1.it (A.I.); federica.palombarini@uniroma1.it (F.P.); alberto.boffi@uniroma1.it (A.B.)

**Keywords:** resveratrol, red wine, ethylchloroformate, gas chromatography–mass spectrometry

## Abstract

Resveratrol (3,5,4′-trihydroxystilbene) is a natural compound that can be found in high concentrations in red wine and in many typical foods found in human diet. Over the past decades, resveratrol has been widely investigated for its potential beneficial effects on human health. At the same time, numerous analytical methods have been developed for the quantitative determination of resveratrol isomers in oenological and food matrices. In the present work, we developed a very fast and sensitive GC–MS method for the determination of resveratrol in red wine based on ethylchloroformate derivatization. Since this reaction occurs directly in the water phase during the extraction process itself, it has the advantage of significantly reducing the overall processing time for the sample. This method presents low limits of quantification (LOQ) (25 ng/mL and 50 ng/mL for *cis*- and *trans*-resveratrol, respectively) and excellent accuracy and precision. Ethylchloroformate derivatization was successfully applied to the analysis of resveratrol isomers in a selection of 15 commercial Italian red wines, providing concentration values comparable to those reported in other studies. As this method can be easily extended to other classes of molecules present in red wine, it allows further development of new GC–MS methods for the molecular profiling of oenological matrices.

## 1. Introduction

**Figure 1:**
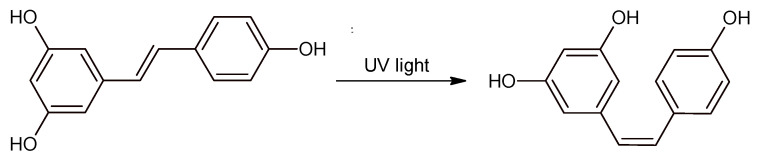
*trans*-RSV to *cis*-RSV isomerization induced by UV light.

Resveratrol (RSV) is a natural compound that can be found in high concentrations in red wine and in many foods found in the human diet [1,2,3,4]. For many years, this molecule has been the subject of many studies concerning its potential benefits for human health. It was shown that RSV is an excellent antioxidant [5], and it can have effects on many cellular processes, from aging and inflammation to stress resistance and cell survival [6,7,8,9]. At the same time, more and more analytical methods have been developed for the quantitative analysis of RSV isomers in oenological and food matrices as well as in biological fluids [10]. Most of these methods are based on HPLC or UPLC coupled with electrochemical, mass spectrometric, or photometric detectors; GC–MS; and capillary electrophoresis [11,12,13,14]. Developing an analytical method for both RSV isomers may be challenging because only *trans*-RSV is commercially available. Typically, *cis*-RSV standard is produced by exposing *trans*-RSV to UV rays (Figure 1).

Our research team has recently developed an analytical method for the simultaneous analysis in red wine of TBDMS derivatives of both RSV isomers and 2,4,6-trihydroxyphenanthrene, a RSV derivative which can be detected in red wine following exposure to UV rays [13,15]. In the present work, we developed a particularly fast and highly sensitive GC–MS method for the analysis of RSV in the form of ethoxycarbonyl derivative. Ethylchloroformate (ECF) derivatization has been known for a long time [16] and has been applied to different classes of molecules including amino and fatty acids, polyamines, and phenolic acids [17,18,19]. Surprisingly, to the best of our knowledge, it has never been applied to the qualitative and quantitative analysis of RSV isomers. Considering that the derivatization reaction with chloroformates occurs in the presence of water during the extraction process itself, this technique could be advantageous for the analysis of RSV because it significantly reduces the processing times of the sample, thus limiting the formation of artifacts. Unlike the classic GC–MS derivatization techniques, which very often require incubation at high temperatures for variable times, ECF can react almost immediately at room temperature with the molecules to be derivatized.

In the present work, we report for the first time the typical mass spectra of the isomers of RSV in the form of ethoxycarbonyl derivatives. We also show that this type of derivatization/extraction is suitable for the development of an analytical method with excellent characteristics of accuracy, precision, linearity, and sensitivity. Once validated, the analytical method will be used for the analysis of RSV in a selection of Italian red wines.

## 2. Results and Discussion

### 2.1. Derivatization Process

**Figure 2:**
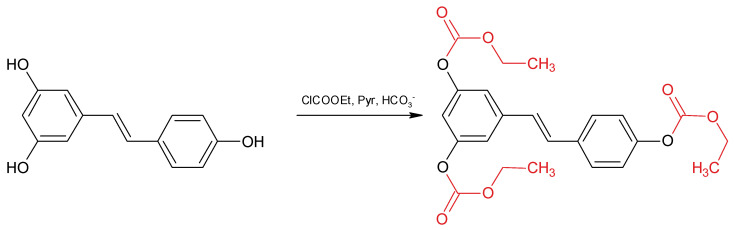
Resveratrol derivatization with ethylchloroformate.

In the present paper, we set up a fast and practical analytical method for the determination of RSV isomers in red wine using ECF as a derivatizing agent (Figure 2).

**Figure 3:**
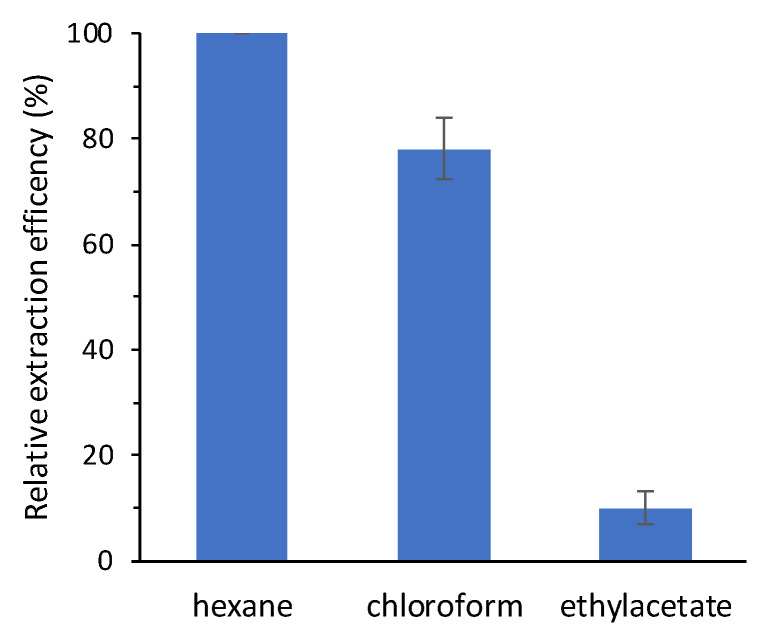
Relative extraction efficiency of derivatized *trans*-RSV with different organic solvents. Relative extraction efficiency of the organic solvents was obtained by setting the highest *trans*-RSV: methyl heptadecanoate peak area ratio equal to 100% (*n* = 3; mean ± SD).

Chloroformates are well known as efficient derivatizing reagents that are able to react in aqueous media, shortening the time required for sample processing. This derivatization methodology requires the use of pyridine as a catalyst, as well as the use of an alkaline environment that allows the ethoxycarbonylation of the phenolic hydroxyl groups. The development of the derivatization conditions was done only with *trans*-RSV. This allows to evaluate any *cis* isomerization inherent in the procedure itself, thus excluding the occurrence of artifacts in the analysis of wines. The first step in developing the method was the optimization of the extraction and of ECF concentration. The solvents generally used in this procedure are either hexane or more polar solvents such as chloroform and ethylacetate [17,18,19]. Therefore, we decided to evaluate the derivatization/extraction efficiency *trans*-RSV with these three solvents using methyl heptadecanoate. This molecule is completely soluble in these three solvents used to test the derivatization/extraction efficiency and it does not have functional groups that can react with ECF, so its concentration does not vary in all phases of the derivatization/extraction process. For this purpose, we extracted 0.5 mL of alkalinized wine with 2 mL of solvent containing methyl heptadecanoate in the presence of a fixed quantity of ECF (20 μL) and pyridine (10 μL) as a catalyst. The results obtained show that extraction with hexane provides the best recovery yields (Figure 3).

By setting hexane as the solvent of choice, we then assessed the amount of ECF to be used. The derivatization efficiency was the same for quantities tested (10, 20, 30, 40, and 50 μL). Eventually, 30 μL was chosen as model concentration, considering that there may be wines richer in RSV (or in other molecules with similar derivatization potential) that could require a higher ECF concentration.

Given the complexity of the oenological matrix, we assessed whether introducing a second extraction step with chloroform could improve the efficiency of the process. At the same time, we decided to perform this second extraction by adding an additional 20 μL of ECF. This further step improves the overall extraction process (+15.33% ± 3.59%; *n* = 3; mean ± SD). However, chloroform extraction has two main drawbacks: the organic phase, representing the bottom layer, is more difficult to recover while an insoluble material is deposited at the interface, which makes quantitative recovery of the organic phase challenging. For this reason, it was decided to reduce the effect of the matrix by extracting half of the starting volume of wine (0.25 mL instead of 0.5 mL). Surprisingly, the yields have doubled, probably due to the extremely favorable organic solvent/aqueous phase ratio (8:1 vs. 4:1).

Since red wine contains on average 13% ethanol, we evaluated whether the extraction/derivatization process was affected by an increase in its concentration. It is known that ethanol is used to promote the formation of ethyl esters of carboxylic acids when ECF is used. Indeed, we have observed that the extraction/derivatization process of molecules bearing carboxylic groups (e.g., gallic acid) is greatly influenced by the concentration of ethanol (data not shown). This is different than in the case of RSV that lacks carboxyl groups. To confirm this assumption, we tested the extraction/derivatization of wine samples in the presence of a higher concentration of ethanol and we observed that, as expected, the derivatization yield remained identical.

### 2.2. GC–MS Characterization of ECF Derivatives

**Figure 4:**
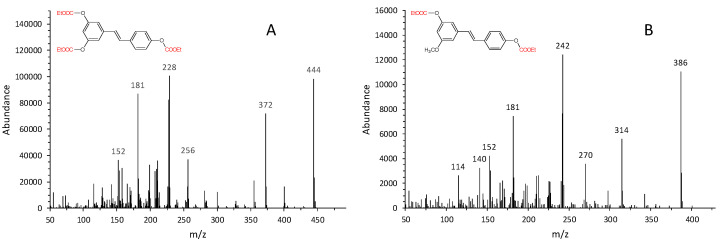
Electron ionization (EI) mass spectra of (**A**) *trans*/*cis*-resveratrol as tri-ethoxycarbonyl derivatives (the isomers show an identical fragmentation pattern) and (**B**) pinostilbene as di-ethoxycarbonyl derivative (internal standard).

Derivatization with ECF makes the molecules particularly suitable for gas chromatographic analysis. RSV isomers and pinostilbene (in the form of ethoxycarbonyl derivatives) are well separated on the HP5–MS chromatographic column with the following retention times: 17.6 min for *cis*-RSV, 18.5 min for pinostilbene (internal standard), and 21.7 min for *trans*-RSV. Furthermore, ethoxycarbonylation of hydroxyl groups is quantitative as no peaks related to partially derivatized species can be detected. In Figure 4, the mass spectra of the ethoxycarbonyl derivatives obtained are reported. The molecular ion is present in the mass spectra of all derivatized species. In addition, a prominent peak corresponding to [M-73]^+^ ion is detected, which corresponds to the ion formed from the loss of ethoxycarbonyl radical (CO_2_C_2_H_5_) form the molecular ion (M^+^). In addition, ions corresponding to the molecular weight of underivatized molecules are always present (*m*/*z* 228 for *cis*- and *trans*-RSV which show the same fragmentation pattern; *m*/*z* 242 for pinostilbene). Given their abundance, those ions were chosen for the validation of the analytical method.

### 2.3. Method Validation

Given the complexity of red wine and the variability of its composition, in our previous work, we developed a wine-like matrix that contains its main constituents [13]. The same matrix was used in the present work as well. In particular, the validation of the analytical method was performed using a pH 3.3 solution containing 13% ethanol and 0.3% *v*/*v* tartaric acid. We opted for this value, as it is reported that in musts from grapes produced from vineyards located in northern regions, the concentration of tartaric acid is higher than 6 g/L, while in musts from southern regions, that concentration does not exceed 2–3 g/L [20]. Therefore, we chose an average value of 4 g/L.

The matrix effect was evaluated for both RSV isomers by comparing the slopes of regression lines in wine-like matrix with the slopes calculated for each isomer in the control wine sample. The experiments were performed in triplicate and the slopes obtained for *trans*-RSV were 0.0033 ± 5.55 × 10^−5^ in wine-like matrix and 0.0034 ± 1 × 10^−4^ in red wine. These values do not significantly differ as assessed by Student’s *t*-test (*p* = 0.649). Similar results were obtained for *cis*-RSV (0.0126 ± 3.06 × 10^−4^ in wine-like matrix vs 0.0126 ± 1.53 × 10^−4^ in red wine; *p* = 1.00). Based on these results, the calibration obtained with wine-like matrix can be used for quantification purposes. In addition, the lack of detectable matrix effect can be explained by the lack of interfering peaks at the retention times of derivatized *trans*- and *cis*-RSV.

The linearity of the method was tested separately on *trans*- and *cis*-RSV. For both of the analytes, a good linearity was achieved (Table 1) with an *R*^2^ coefficient always ≥0.999.

Limit of quantification (LOQ) corresponds to the lowest concentration value used in the calibration plot, i.e., 50 ng/mL for *trans*-RSV and 25 ng/mL for *cis*-RSV. At lower concentrations, at S/N = 3, it was not possible to identify RSV isomers in a reliable way. Thus, in this specific case, LOQ and limit of detection (LOD) values are the same. The same experimental observation was reported also by Paulo et al. [21].

In comparison to all protocols that require one or more extraction steps with organic solvents followed by a derivatization step, the use of ECF has the advantage of directly derivatizing the molecules in the presence of the aqueous phase while the extraction process is taking place. This allows a considerable reduction of the sample’s processing times while ensuring almost complete substrate recovery. At least two consecutive extraction/derivatization steps were needed to fully recover the analytes from wine-like matrix. As reported in Table 1, the recovery of each RSV isomer at two different concentrations (*trans*-RSV: 200 ng/mL and 2000 ng/mL; *cis*-RSV: 100 ng/mL and 1000 ng/mL) was >99%. Concerning precision, the % RSD values obtained both for *trans*- and for *cis*-RSV fell well within the criteria normally accepted in bioanalytical method validation being lower than 10% [22].

### 2.4. Red Wine Analysis

Red wine has been consumed by humans for hundreds of years and its beneficial effects on human health are well described [23,24]. The antioxidant activity of red wine is due to the synergy of *cis*- and *trans*-RSV with other molecules such as catechins, anthocyanins, polyphenols, and flavanols, which are particularly abundant in this specific oenological matrix [25]. In the early 1990s, RSV became popular as it was recognized as one of the main components of red wine responsible for the so-called French paradox, according to which the French have a low incidence of coronary heart disease despite consuming a diet rich in saturated fats [24]. Since then, several GC–MS analytical methods have been developed for the quantitative analysis of RSV isomers in red wine [14]. Since both RSV isomers show remarkable antioxidant properties [26], it is essential to determine also the *cis* isomer, which is present in non-negligible quantities in wine. The method validated in this work, unlike the others, has the advantage of being particularly fast as the derivatization with the ECF proceeds directly in the aqueous phase at room temperature. This method was applied to the quantitative analysis of the RSV isomers in 15 wines from different Italian regions that differ in vintage and grape variety.

**Figure 5:**
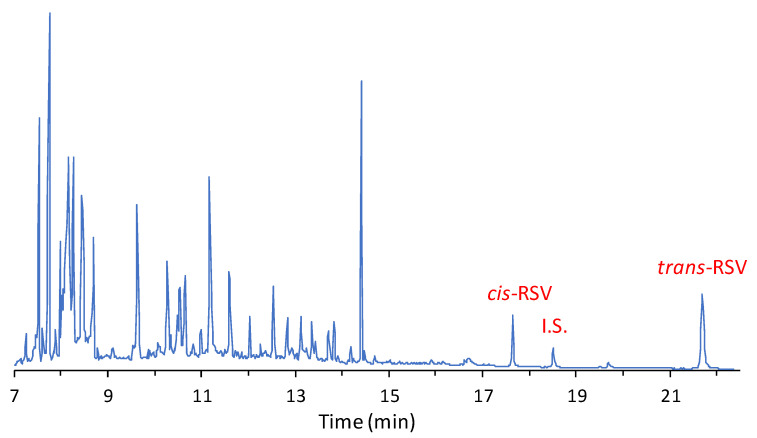
Typical GC–MS chromatogram of a red wine sample derivatized with ethylchloroformate.

In Figure 5, a typical GC–MS chromatographic profile of a wine sample submitted to ECF derivatization is reported.

RSV isomers and the internal standard are well resolved and elute in a part of the chromatogram free of interfering peaks. In addition, in the first 15 min of elution, it is possible to observe the presence of numerous peaks, among which there are molecules with acidic functional groups that are derivatized as ethyl esters (e.g., gallic acid which elutes at 14.4 min). This reaction is possible as about 13% ethanol is normally present in red wine. As expected, the quantitative analysis shows that RSV content can vary significantly between wines (Table 2).

This parameter is influenced mainly, but not exclusively, by the grapes that are used for red wine production, as RSV is found in widely varying amounts among grape varieties. For example, it is known that the grape variety known as “pinot noir” is particularly rich in RSV as well as the wine derived from it [27]. In the present work, we obtained a similar result in that, among the wines tested, pinot noir (#3) shows the highest concentration of both *trans*- and *cis*-RSV.

Overall, the total RSV content in the 15 wines tested in this paper ranges from a minimum of 336.42 ng/mL to a maximum of 3095.70 ng/mL. These data are comparable with those reported in other papers, where RSV was determined with different analytical methods on red wines from different geographical origin [13,21,26].

In addition, two of the wines analyzed in this study (#6 and #11) had already been analyzed with a different analytical method developed by our research group [13]. This method, which involved extraction with organic solvents and derivatization with TBDMS, had provided *trans*- and *cis*-RSV values equal to 266.4 ng/mL and 77.6 ng/mL, respectively, for wine #11, and 848.2 ng/mL and 283.7 ng/mL, respectively, for wine #6. These data are comparable to those reported in Table 2 with a variation in total RSV content lower than 4%. These data further confirm the accuracy of the analytical method here developed.

In conclusion, we have set up an analytical method for the analysis of RSV in red wines based on ECF derivatization. This method is fast, sensitive, and specific, providing low LOD and LOQ. Precision and accuracy are in conformity with the criteria normally accepted in methods validation, with practically total recovery and a percentage RSD lower than 5%.

Finally, this work demonstrates that derivatization with ECF can also be extended to other classes of molecules present in wine such as polyphenols. We have, in fact, observed that by varying the concentration of ethanol during the extraction step, it is possible to optimize the analysis of phenolic acids such as gallic acid as well. This provides a future blueprint for the development of new analytical methods in GC–MS aimed at the molecular characterization of oenological matrices.

## 3. Materials and Methods

### 3.1. Reagents and Standards

Hexane, chloroform, ethylchloroformate, *trans*-RSV, and pinostilbene (internal standard) were purchased from Sigma-Aldrich (Germany). Standard stock solutions were prepared by dissolving *trans*-RSV and pinostilbene in ethanol. All calibrations were performed by diluting the stock solutions in wine-like matrix (50 mL final volume containing: 150 mg disodium tartrate, 6.5 mL ethanol (13% final concentration), 43.5 mL H_2_O adjusted to pH 3.3 with an aqueous tartaric acid solution). *cis*-RSV standard solution was prepared according to Francioso et al. [13]. Briefly, 30 μg/mL *trans*-RSV in wine-like matrix were exposed for 2 min to UV light (20 cm from the irradiation source, with a 14.7 W UV-B fluorescent tube emitting at wavelengths of 270−320 nm with a peak at 312 nm). Conversion rate was estimated using GC–MS by comparing *trans*-RSV peak areas before and after the treatment with UV light.

### 3.2. Extraction/Derivatization Procedure

RSV isomers were subjected to derivatization with ECF according to the following protocol: 0.25 mL of wine sample or standards dissolved in wine-like matrix (containing 500 ng of pinostilbene) were put in a 10 mL glass tube. This tube was shielded from light by wrapping it with aluminum foil to minimize light-induced isomerization of *trans*-RSV. The solution was made alkaline (pH > 9) by adding 65 μL of 0.6 M NaHCO_3_. Hexane (2 mL) and ECF (30 μL) were added to this solution followed by the slow addition of 10 μL of pyridine as catalyst. The tube was left uncapped for a few seconds to allow the releasing of CO_2_. After 2 min shaking, the organic layer was removed, and a second extraction step was performed with chloroform (2 mL) containing further 20 μL of ECF. The lower organic layer was removed, combined with the hexane extract, and dried using a nitrogen stream. The sample was resuspended in 75 μL of chloroform and subjected to GC–MS analysis.

Extraction efficiency of derivatized *trans*-RSV was tested with three different organic solvents (hexane, chloroform, or ethylacetate) was tested in the same wine sample using methyl heptadecanoate as an internal standard. Methyl heptadecanoate was dissolved in the solvents used for the extraction at a final concentration of 500 ng/mL. Relative derivatization efficiency of the organic solvents was obtained by setting the highest *trans*-RSV: methyl heptadecanoate peak area ratio equal to 100%.

### 3.3. Gas Chromatography–Mass Spectrometry

GC–MS analyses were performed with an Agilent 7890B gas chromatograph coupled to a 5977B quadrupole mass selective detector (Agilent Technologies, Palo Alto, CA, USA). Chromatographic separations were carried out with an Agilent HP5ms fused-silica capillary column (30 m × 0.25 mm i.d.) coated with 5% phenyl 95%-dimethylpolysiloxane (film thickness 0.25 μm) as stationary phase. Injection mode: splitless at a temperature of 260 °C. Column temperature program: 70 °C (1 min) then to 300 °C at a rate of 15 °C/min and held for 5 min, solvent delay: 7 min. The carrier gas was helium at a constant flow of 1.0 mL/min. The spectra were obtained in the electron impact mode at 70 eV ionization energy; ion source 280 °C; ion source vacuum 10^−5^ Torr. Mass spectrometric analysis was performed simultaneously in TIC (mass range scan from *m*/*z* 50 to 650 at a rate of 0.42 scans s^−1^) and SIM mode (selected ions: *m*/*z* 242 for internal standard and *m*/*z* 444 for *cis*/*trans*-RSV).

### 3.4. Method Validation

Calibrations were performed adding increasing amounts of RSV isomers to 0.25 mL of wine-like matrix containing 500 ng of pinostilbene as internal standard. The calibration samples were subjected to the ECF derivatization as described above.

Calibration plot for *trans*-RSV was performed in the range of 50–3000 ng/mL (seven calibration points). Calibration curve of *cis*-RSV standard was performed in a separate experiment in the range of 25–1000 ng/mL (six calibration points). *cis*-RSV standard was obtained at a final concentration of 10 μg/mL by exposing *trans*-RSV (30 μg/mL in wine-like matrix) to UV light for 2 min. Three replicate analyses were performed at each concentration in wine-like matrix. The calibration curves were obtained by plotting the peak area ratio between each analyte and the internal standard versus analyte concentration.

Accuracy and precision were determined in a wine sample spiked with *trans*-RSV and *cis*-RSV at two different final concentrations (*trans*-RSV: 200 ng/mL and 2000 ng/mL; *cis*-RSV: 100 ng/mL and 1000 ng/mL) analyzing five replicates for each concentration in the same day. Spiked and unspiked wine samples were subjected to ECF derivatization and analyzed by GC–MS.

Accuracy was evaluated through standard recovery experiments. A comparison of the amount found versus the amount added provides the recovery of the method (%) which is an estimate of the accuracy of the method itself. The same samples reported above were also used to determine method precision expressed as % relative standard deviation (% RSD).

LOD and LOQ were determined by the analysis of wine-like matrix with decreasing concentrations of *trans*-RSV and *cis*-RSV. The limit of detection (LOD) of the target compounds is taken at S/N = 3, whereas the limit of quantification (LOQ) was set to S/N = 10.

The matrix effect was evaluated by analyzing *trans*-RSV and *cis*-RSV both in wine-like matrix and in red wine, and by comparing the slopes of the regression plots by Student’s *t*-test.

### 3.5. Red Wine Analysis

*trans*-RSV and *cis*-RSV were measured in fifteen different red wines from different Italian regions, grape varieties, and vintage. 0.25 mL of each red wine were spiked with 500 ng of pinostilbene as internal standard and submitted to derivatization/extraction procedure as described above.

## Figures and Tables

**Table 1:** Validation parameters.

Compound	Range (ng/mL)	Slope	Intercept	*R* ^2^	LOQ (LOD) * (ng/mL)	Concentration (ng/mL)	Accuracy (recovery %)	Precision (RSD %)
***trans*-RSV**	50–3000	0.0033	0.0907	0.9992	50	200	99.02	5.46
						2000	99.20	3.28
***cis*-RSV**	25–1000	0.0126	0.08456	0.9991	25	100	103.11	4.19
						1000	99.88	1.58

* LOQ and LOD values are the same for both RSV isomers.

**Table 2:** *trans*- and *cis*-resveratrol content in a selection of Italian red wines (values are the mean of two measurements).

Wine	Vintage	Italian Region	Varieties	*trans*-RSV (ng/mL)	*cis*-RSV (ng/mL)	Total RSV (ng/mL)
#1	2018	Piemonte	100% Barbera	1185.06	343.27	1528.33
#2	2019	Alto Adige	100% Lagrain	475.97	170.77	646.74
#3	2017	Alto Adige	100% Pinot Noir	1772.94	1322.76	3095.70
#4	2017	Veneto	70% Corvina, 30% Rondinella	275.97	67.84	343.81
#5	2015	Friuli Venezia Giulia	100% Cabernet Franc	766.88	302.48	1069.36
#6	2016	Toscana	90% Sangiovese, 10% Merlot	885.06	290.44	1175.50
#7	2016	Toscana	100% Sangiovese	1339.61	688.73	2028.34
#8	2018	Umbria	70% Sangiovese, 15% Merlot, 15% Sagrantino	688.09	145.98	834.07
#9	2018	Lazio	100% Cesanese	385.06	156.88	541.94
#10	2019	Lazio	100% Cabernet Sauvignon	594.15	154.67	748.82
#11	2016	Campania	100% Aglianico	254.76	81.66	336.42
#12	2016	Puglia	100% Primitivo	891.12	243.44	1134.56
#13	2018	Puglia	100% Negramaro	945.67	408.77	1354.44
#14	2018	Sicilia	60% Merlot, 40% Cabernet Sauvignon	485.06	123.66	608.72
#15	2018	Sicilia	100% Syrah	530.52	213.86	744.38
